# Gynecomastia What the Surgeon Needs to Know

**Published:** 2009-01-15

**Authors:** Carol J. Singer-Granick, Mark S. Granick

**Affiliations:** ^a^ Department of Pediatrics, Division of Endocrinology; ^b^ Department of Surgery, Division of Plastic Surgery, New Jersey Medical School – UMDNJ, Newark, NJ

## Abstract

**Objective:** The purpose of this review is to present the complex underlying pathophysiology that can form the basis of this common condition. **Methods:** More than 20 years of clinical experience in endocrinology and plastic surgery and a review of the English-language literature were used to form the basis of this review. **Results:** While idiopathic and physiologic causes are frequent, there are multiple, significant, underlying disorders that can result in gynecomastia, including chronic illness, cancer, medications, syndromes, and a variety of endocrinopathies. **Conclusion:** Both history and physical examination are frequently sufficient to make an appropriate diagnosis. In patients who do not have a definitive etiology of their gynecomastia, a screening battery of laboratory tests is sufficient to rule out significant pathophysiology, although these tests may be difficult to interpret in children and adolescents. An endocrinology consultation is suggested whenever an abnormal screening laboratory test occurs or if there are any other suggestions of underlying endocrinopathy.

## DESCRIPTION

*Gynecomastia* refers to the condition of breast development in a male. It can occur in boys and men of all ages and is most common in infancy and adolescence and in middle-aged to older men. The pathophysiology of gynecomastia is felt to be an imbalance of estrogens and androgens, with a decreased testosterone-to-estradiol ratio.^1^ This imbalance can occur through many mechanisms and directly affects breast tissue.^2^ Transient gynecomastia is estimated to occur in 60% to 90% of male infants secondary to high estrogen state during pregnancy.^3^,^4^ Pubertal gynecomastia has a peak prevalence of nearly 65% and occurs at about 14 years of age.^3^,^5^ Older men also develop involutional gynecomastia, with a prevalence of 40% to 55%, seen at autopsy.^6^ Frequently, the etiology of gynecomastia is evident when a thorough history and physical examination are performed. When the etiology is not apparent, then a series of laboratory tests should be performed to rule out significant underlying pathophysiology.

## PATHOPHYSIOLOGY

Gynecomastia is associated with a host of conditions as well as numerous additional etiologies (Tables 1 and 2). The altered ratio of estrogens to androgens or increased breast sensitivity to normal circulating estrogen levels results in ductal hyperplasia, elongation, and branching, along with fibroblast proliferation and increased vascularity.^7^–^10^ Males produce estrogen primarily from converting peripheral androgens, testosterone and androstenedione, to estradiol and estrone via the aromatase enzyme. This occurs mainly in muscle, fat, and skin. In a male adult, the normal production ratio of androgen to estrogen is 100:1. In the circulation, the ratio of testosterone to estrogen is 300:1.^11^

**Figure 1:**
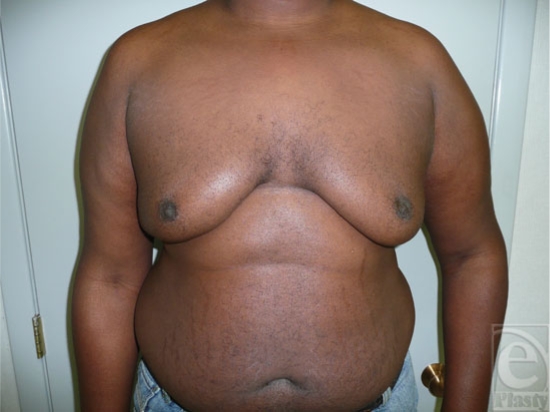
Obese patient with fatty breasts and enlarged breast nodules.

**Figure 2:**
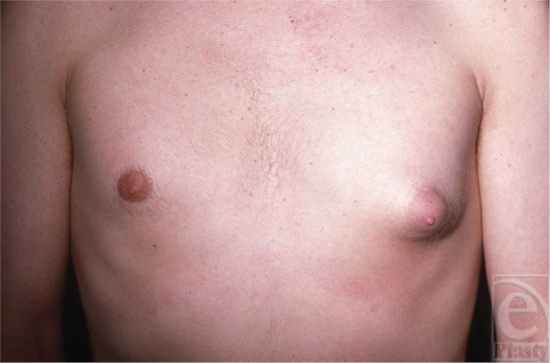
Asymmetric breast development.

It is sometimes difficult to differentiate between fatty tissue and breast tissue, especially in overweight individuals (Fig 1). *Gynecomastia* is clinically defined by the presence of a rubbery, firm mass extending concentrically from the nipples. This subareolar disk of glandular tissue has been described as feeling like a corded rope.^5^ In contrast, *pseudogynecomastia* is defined as the proliferation of soft subcutaneous fat that can give males the appearance of developing breasts. Breast development can be unilateral or bilateral and asymmetry is often an early stage in the development of gynecomastia (Fig 2).

The histology associated with gynecomastia is related more to the duration than the cause of the process. When the condition is asymptomatic, it has frequently existed for months or years prior to presentation. Chronic changes include dilated ducts with periductal fibrosis, stromal hyalinization, and increased subareolar fat. Those presenting with pain and tenderness frequently have breast hypertrophy of more recent onset. In contrast, their pathologic studies show ductal hyperplasia with inflammation of the periductal tissue and subareolar fat.^6^–^10^

**Figure 3:**
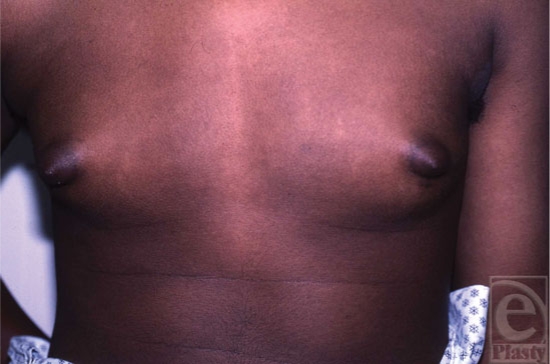
Pubertal gynecomastia.

*Pubertal gynecomastia* refers to the transient condition of breast development occurring in 10- to 16-year-old boys (Fig 3). About 40% of boys develop this condition, which peaks at 14 years of age at nearly 65% incidence. These statistics are based on clinical studies detecting breast enlargement as small as 0.5 cm in diameter. About 10% of boys report gynecomastia in general surveys of sexual development.^5^ Some investigators have found brief elevations of plasma estradiol in some affected male adolescents, but sustained elevations of estrogens are not present in pubertal gynecomastia.^1^,^12^ The pathogenesis of pubertal gynecomastia appears to be an elevated conversion of adrenal androgens to estrogens during the daytime when testosterone secretion is low.^5^,^12^ In the early stages of puberty, testosterone secretion occurs primarily at night and with pubertal progression, circulating gonadotropins and testosterone levels begin to rise during the day. Estrogens, which are primarily from adrenal androgens, in contrast, start to rise throughout the 24-hour daytime period in early adolescence. In contrast, adrenal androgens may suppress breast formation during the daytime in some boys. Boys with pubertal gynecomastia show decreased adrenal androgen-to-estrogen ratio in the afternoon compared with unaffected boys.^13^ Either decreased adrenal production of androgens or increased aromatization causes this transient pubertal gynecomastia. Increased aromatase activity has been reported in the skin fibroblasts of boys affected with gynecomastia, whereas it is not seen in unaffected boys.^14^

**Figure 4:**
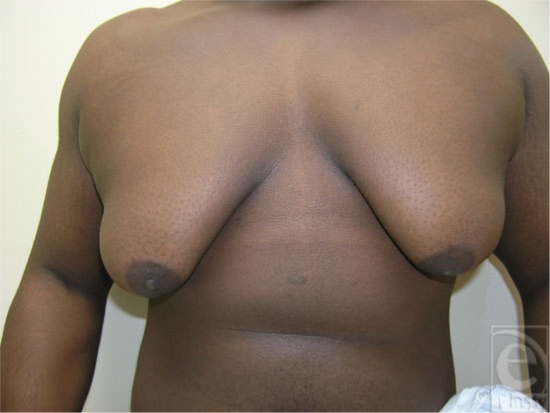
Macrogynecomastia with female-like breast size and shape.

Boys showing pubertal gynecomastia usually have breast tissue measuring less than 4 cm. Signs of development of male characteristics generally precede the gynecomastia and the adolescent is usually Tanner stages II to III.^5^ In most cases, the condition resolves in 1 to 3 years. In 75% of boys, the condition disappears in 2 years, and 90% resolve within 3 years. Persistent gynecomastia is seen in less than 5% of affected boys. However, the term “macrogynecomastia” has been used to describe males with glandular tissue exceeding 5 cm in diameter and the breasts resembling female breasts Tanner stage IV with secondary mound formation (Fig 4). This condition rarely regresses spontaneously. Obese individuals may achieve a decrease in glandular tissue or its prominence by reducing their weight.^15^ Recently, prepubertal gynecomastia, which is a rare condition and often pathologic, was found to be associated with lavender and tree tea oils.^16^ The 2 oils were found to have estrogenic and antiandrogenic activities. The condition resolved soon after the boys discontinued the topical application. Another recent report of prepubertal gynecomastia came from Germany where a large family was studied showing dominant transmission of prepubertal gynecomastia over 3 generations.^17^ It was found that a repeat polymorphism of the *P450* aromatase gene cosegregated with the disease phenotype. Excess serum and normal estradiol levels were present, causing prepubertal gynecomastia and hypogonadism in the boys and the men.

**Figure 5:**
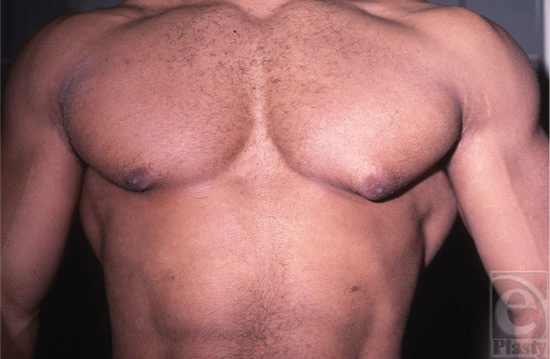
Gynecomastia in an athlete who had taken anabolic steroids.

Gynecomastia in adults is frequently multifactorial, resulting from decreased testosterone production in the aging testes, increased aromatization of androgens to estrogens as the body fat increases, and often medications that are more likely taken by older individuals (Table 3).^3^,^11^ Medicines implicated in causing gynecomastia include estrogens or drugs with estrogen-like activity (Fig 5); antiandrogens or inhibitors of androgen synthesis; and drugs acting by unknown mechanisms, such as psychoactive drugs, cytotoxic agents, cardiovascular agents, antiulcer medications, antibiotics, and antiviral therapeutics.

**Figure 6:**
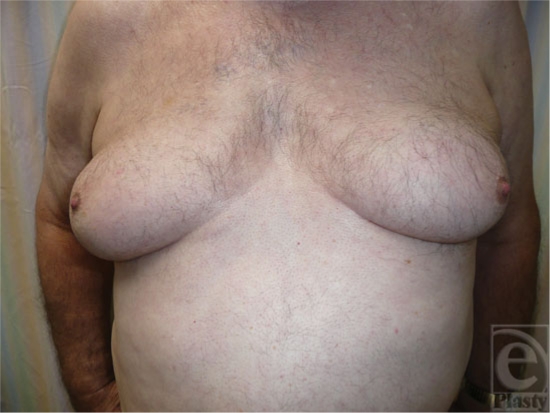
Gynecomastia in a patient with chronic liver disease.

Most patients presenting to a physician for the evaluation of gynecomastia have either an idiopathic condition or persistent pubertal gynecomastia (Table 2).^3^ Although physiologic gynecomastia is more common than pathologic causes, these other etiologies cannot be overlooked. Conditions causing hypogonadism and decreased androgen production and/or action include Klinefelter syndrome, Kallmann syndrome, Reifenstein syndrome, congenital anorchia, testicular trauma or torsion, viral orichitis, pituitary tumors, malignancies increasing human chorionic gonadotropins (hCGs), renal and liver failure (Fig 6), hyperthyroidism, malnutrition, androgen insensitivity and 5-*α*-reductase deficiency, and other forms of congenital adrenal hyperplasia.^3^,^11^ Alcohol causes increased testosterone clearance from the circulation through enhanced hepatic A-ring reductase activity.^6^ Also, several medications such as spironolactone and ketoconazole inhibit testosterone synthesis.

**Figure 7:**
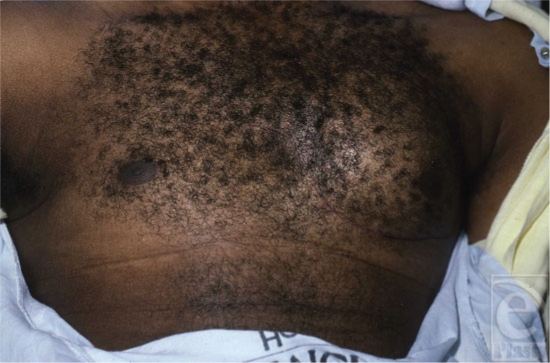
Left-side breast cancer presenting in a male with asymmetric breasts.

The increased production or action of estrogens can occur peripherally or at the testicular level.^3^,^11^ Testicular tumors producing estrogen or the ectopic production of hCG such as in the lung and the kidneys and gastrointestinal germ cell tumors can account for increased estrogens. In addition, peripheral conversion secondary to increased substrate or increased activity of the aromatase enzyme can lead to excessive estrogens. Increased aromatization occurs during aging, reflecting the relative increase in body fat. Excess estrogens also can occur in malnutrition, hyperthyroidism, adrenal tumors, and chronic liver and renal diseases. Hyperestrogenic states can decrease androgen secretion by suppressing pituitary luteinizing hormone, which subsequently suppresses testicular testosterone secretion. High estrogen levels also inhibit cytochrome P450c17 enzymes in the testes, which are necessary for testosterone synthesis. The patient's medication, alcohol use, drug use, smoking habit, and food history are critical. Other conditions to consider in the differential diagnosis of breast enlargement, especially unilateral, include dermoid cyst, neurofibroma, lymphangioma, hematoma, lipoma, breast cancer and metastases from neuroblastoma, lymphocytic leukemia, lymphoma, and rhabdomyosarcoma. Testicular tumors including Sertoli tumors, Leydig cell tumors, and germ cell tumors can also lead to gynecomastia. Although accounting for less than 1% of male cancers, an accurate diagnosis is critical to determine breast cancer. It usually presents as a unilateral, eccentric mass that is frequently firm or attached to underlying tissue (Fig 7). There may also be nipple crusting or discharge and lymphadenopathy in the axillae.^5^

Patients frequently present to the surgeon before being evaluated by an endocrinologist. What evaluation is necessary to distinguish pathologic from physiologic conditions? It is important to obtain a medication history, a detailed family history, and a complete medical history to identify cirrhosis, renal failure, hyperthyroidism, other hormonal abnormalities, malnutrition, and chest wall trauma. Male adolescents with breast tissue less than 4 cm in diameter generally need only reassurance and follow-up. Otherwise, laboratory tests should include renal, hepatic, and thyroid function studies if indicated by history or physical examination (Table 4). Boys who appear to have hypogonadism, macrogynecomastia, or precocious puberty should undergo determinations of luteinizing hormone, follicle-stimulating hormone, estradiol, dehydroepiandrosterone sulfate, and hCG. If galactorrhea is present, a prolactin level should be obtained. If any of these tests are abnormal, an endocrinologist should be consulted. If an individual has hard lymph nodes, dimpling of the skin, and/or breast discharge or bleeding, a malignancy must be considered. When considering breast cancer, both fine-needle biopsy and mammogram are indicated.

## MEDICAL TREATMENT

The physician should treat the specific condition causing gynecomastia, if one is identified. Medications that cause gynecomastia should be discontinued. Softening of the glandular tissue along with decreased tenderness will usually occur by 1 month. However, if breast development has been present for more than 1 year, it rarely regresses substantially because of fibrosis. Hypogonadism of various causes can be treated with testosterone, and regression of gynecomastia should occur if the condition is short term.

Although medical therapy with estrogen antagonists has not been approved for the treatment of gynecomastia, various researchers have shown different levels of effectiveness for the regression of both the pain and size of the breast tissue. A retrospective analysis comparing danazol with tamoxifen was done on 43 patients with idiopathic gynecomastia, ranging in age from 13 to 82 years (mean = 39.5 years).^18^ The median size of the breast tissue was 3 cm and the median duration of gynecomastia was 3 months. Of the 23 patients who were treated with tamoxifen, complete resolution occurred in 18 (78.2%), whereas 8 of the 20 (40%) who were treated with danazol had complete resolution. A decrease in pain occurred in 82% of the patients in the tamoxifen group and 75% in the danazol group. However, 5 patients in the tamoxifen cohort developed recurrences of the breast mass. There was progressive enlargement in 1 patient in each of the groups undergoing medical treatment. No adverse effects were seen except for 1 male developing calf muscle pain, which was shown not to be due to thrombosis.

A prospective study on the use of tamoxifen in physiologic gynecomastia was conducted in 36 men who were classified as having either lump or fatty gynecomastia.^19^ *Lump gynecomastia* was defined as a single palpable solid lesion in the retroareolar region and was observed in 20 men. Sixteen men had *fatty gynecomastia*, referring to those presenting with breast enlargement and no palpable solid lump. The patients ranged in age from 18 to 64 years (mean = 31 years), and the mean duration of gynecomastia was 4 months. Pain and tenderness were characterized in 25 cases. The patients took tamoxifen for a mean of 12 weeks (duration = 4–24 weeks). Resolution of the mass occurred in 83.3% of the total patients, 100% in the lump group and 62.5% in the fatty group. Tenderness decreased in 84% of the total patients, 100% in the lump group and 69% in the fatty group. There was only 1 recurrence after 7 months. There were no major adverse effects except for a deep vein thrombosis in a 23-year-old man who sustained major lower limb trauma.

A retrospective chart review compared 38 patients with persistent pubertal gynecomastia assigned to either tamoxifen or raloxifene treatment group.^20^ The mean age was 14 years, and the mean duration of gynecomastia was 28.3 months. Tamoxifen is an antiestrogen that has estrogenic effects in all tissues other than the breast. Raloxifene is a nonsteroidal antiestrogen that shows estrogenic effects for skeletal and lipid tissues and antiestrogenic effects for the breasts and the uterus. The mean diameter of breast nodules decreased by 2.1 cm after tamoxifen treatment and 2.5 cm after raloxifene treatment. Improvement was noted in 86% of the tamoxifen-treated group and 91% in the raloxifene-treated group. No adverse effects were seen in either group. Thus, in spite of the relatively long duration of gynecomastia prior to treatment, a high percentage of patients responded to both treatment modalities. However, because there was no follow-up in untreated patients, the final assessment is murky regarding treatment versus observation alone. In addition, although no treated patients complained of a recurrence, 40% went for surgery because the medical treatment did not completely solve the problem.

Anastrozole, another nonapproved treatment for gynecomastia, was evaluated in a randomized, double-blinded, placebo-controlled study of 80 male adolescents over a 3-month period.^21^ Anastrozole is a potent aromatase inhibitor that decreases estrogen levels and increases testosterone concentration. A *response* was defined as 50% or more reduction in the calculated volume of both breasts by using ultrasonography. In the anastrozole group, there was a 38.5% response versus a 31.4% response in the placebo group. This was not significant. Similarly, a randomized placebo-controlled study comparing tamoxifen with anastrozole was evaluated for the prevention of bicalutamide-induced gynecomastia in 93 men with prostate cancer.^22^,^23^ Men receiving tamoxifen had a significantly reduced risk of gynecomastia compared with the anastrozole-treated group in which the risk was similar to the placebo-treated group. These studies suggest that selective estrogen antagonists may be the pharmacologic treatment of choice for most patients with gynecomastia.

## CONCLUSIONS

Gynecomastia is usually a physiologic condition that may regress over time. The etiology is often determined as a result of both thorough history and physical examination. However, an atypical presentation can signify a pathologic state and the surgeon should be aware of associated problems. If the gynecomastia involves breast tissue of 5 cm or more or if there is an atypical presentation, a hormonal and sometimes radiologic workup should be performed. Suspicious breast lesions should be biopsied. Hormonal studies are frequently difficult to interpret and are based on age and Tanner staging in the adolescents and children. When there is a screening laboratory abnormality or if the etiology is in doubt, an endocrinologist should be consulted. Medical treatments of gynecomastia are currently being studied, and antiestrogens may become the pharmacologic treatment of choice within certain presentations and conditions.

## Figures and Tables

**Table 1:** Conditions associated with gynecomastia^3^,^5^,^6^,^11^

*Physiologic*
Neonatal
Pubertal
Involutional
*Pathologic*
Neoplasms
Testicular
Pituitary
Breast tumors
Adrenal
Liver
Human Chorionic Gonadotropin—ectopic production
Lymphoma/leukemia
Endocrinopathies
Hypogonadism
Syndromes: Klinefelter, Kallman's
Androgen insensitivity
Hermaphroditism
Enzymatic defects of testosterone synthesis
Testicular injury/regression
Hyperthyroidism
High aromatase
Adrenal hyperplasia
Corticotropin deficiency
Chronic Illnesses:
Liver disease
Renal disease
Malnutrition
Cystic fibrosis
AIDS
Ulcerative Colitis
Medications (see Table 3)

**Table 2:** Etiologies of gynecomastia^3^,^11^

Idiopathic gynecomastia (no detectable abnormalitiy)	25%
Pubertal gynecomastia	25%
Secondary to medication	10–20%
Cirrhosis or malnutrition	8%
Primary hypogonadism	8%
Testicular tumors	3%
Secondary hypogonadism	2%
Hyperthyroidism	1.5%
Chronic renal disease	1%

**Table 3:** Medications implicated in gynecomastia^3^,^5^,^11^

Hormones
Estrogens and estrogen agonists
Androgens and anabolic steroids
Human chorionic gonadotropin
Androgen antagonists
Ketoconazole
Flutamide
Metronidazole
Finasteride
Spironolactone
Etomidate
Antiulcer drugs
Cimetidine
Omeprazole
Ranitidine
Cytotoxic agents
Bisulfar
Vincristine
Nitrosoureas
Procarbazine
Cisplatin
Methotrexate
Cyclophosphamide
Chlorambucil
Psychoactive drugs
Tricyclic antidepressants
Phenothiazines
Diazepam
Cardiovascular agents
Amiodarone
Angiotensin-converting enzyme inhibitors
Calcium channel blockers
Digitoxin
Methyldopa
Antituberculotic agents
Ethionamide
Thiacetazone
Isoniazid
Antiviral therapeutics
Protease inhibiters
Miscellaneous
Marijuana
Heroine
Methadone
Alcohol
Amphetamines
Phenytoin
Penicillamine

**Table 4:** Laboratory profile for gynecomastia*

Complete metabolic profile
*β*-Human chorionic gonadotropin
Dehydroepiandrosterone sulfate
Luteinizing hormone/follicle-stimulating hormone
Testosterone
Estradiol
Prolactin
Free thyroxine
Thyrotropin

*Any abnormalities in this profile warrants an endocrinology consult preoperatively.

## References

[1] Lee PA (1975). The relationship of concentration of serum hormones to pubertal gynecomastia. J Pediatr.

[2] Mathur R, Braunstein GD (1997). Gynecomastia: pathomechanisms and treatment strategies. Horm Res.

[3] Braunstein GD (1993). Gynecomastia. N Engl J Med.

[4] Hall PF (1959). Gynaecomastia.

[5] Mahoney CP (1990). Adolescent gynecomastia differential diagnosis and management. Pediatr Clin N Am.

[6] Braunstein GD (2007). Gynecomastia. N Engl J Med.

[7] Williams MJ (1963). Gynecomastia: its incidence, recognition and host characterization in 447 autopsy cases. Am J Med.

[8] Bannayan GA, Hajdu SI (1972). Gynecomastia: clinicopathologic study of 351 cases. Am J Clin Pathol.

[9] Andersen JA, Gram JB (1982). Gynecomasty: histological aspects in a surgical material. Acta Pathol Microbiol Immunol Scand.

[10] Nicolis GL, Modlinger RS, Gabrilove JL (1971). A study of the histopathology of human gynecomastia. J Clin Endocrinol Metab.

[11] Allee MR, Baker MZ Gynecomastia. eMedicine.

[12] Large DM, Anderson DC (1979). Twenty-four profiles of circulating androgens and estrogens in male puberty with and without gynecomastia. Clin Endocrinol.

[13] Moore DC, Schlaepter LV, Paunier L (1984). Hormonal changes during puberty, transient pubertal gynecomastia: abnormal androgen-estrogen ratios. J Clin Endocrinol Metab.

[14] Bullard J, Mowszowicz I, Schaison G (1987). Increased aromatase activity in skin fibroblasts from patients with isolated gynecomastia. J Clin Endocrinol Metab.

[15] Styne DM (2004). Pediatric Endocrinology.

[16] Henley DK, Lipson N, Koruck KS, Bloch CA (2007). Prepubertal gynecomastia linked to lavender and tea tree oils. N Engl J Med.

[17] Binder G, Iliev DI, Dufke A (2005). Dominant transmission of prepubertal gynecomastia due to serum estrone excess: hormonal, biochemical and genetic analysis in a large kindred. J Clin Endocrinol Metab.

[18] Ting ACW, Chow LWC (2000). Comparison of tamoxifen with danazol in the management of idiopathic gynecomastia. Am Surg.

[19] Khan HN, Rampaul R, Blamey PW (2004). Management of physiological gynaecomastia with tamoxifen. Breast.

[20] Lawrence SE, Faught KA, Vethamuthu J, Lawson ML (2004). Beneficial effects of raloxifene and tamoxifen in the treatment of pubertal gynecomastia. J Pediatr.

[21] Plourde PV, Reiter EO, Jou HC (2004). Safety and efficacy of anastrozole for the treatment of pubertal gynecomastia: a randomized, double-blind, placebo-controlled trial. J Clin Endocrinol Metab.

[22] Saltzstein D, Cantwell A, Sieber P (2002). Prophylactic tamoxifen significantly reduces the incidence of bicalutamide-induced gynecomastia and breast pain. Br J Urol Int.

[23] Saltzstein D, Seiber P, Morris T, Gallo J (2005). Prevention and management of bicalutamide-induced gynecomastia and breast pain: randomized endocrinologic and clinical studies with tamoxifen and anastrozole. Prostate Cancer Prostatic Dis.

